# Sirtuins in Neuroendocrine Regulation and Neurological Diseases

**DOI:** 10.3389/fnins.2018.00778

**Published:** 2018-10-26

**Authors:** Yuki Fujita, Toshihide Yamashita

**Affiliations:** ^1^Department of Molecular Neuroscience, Graduate School of Medicine, Osaka University, Suita, Japan; ^2^WPI Immunology Frontier Research Center, Osaka University, Suita, Japan; ^3^Graduate School of Frontier Biosciences, Osaka University, Suita, Japan

**Keywords:** sirtuin, SIRT1, central nervous system, axon degeneration, neuronal development

## Abstract

Silent information regulator 1 (SIRT1) is a mammalian homolog of the nicotinamide adenine dinucleotide (NAD)-dependent deacetylase sirtuin family. Sirtuin was originally studied as the lifespan-extending gene, silent information regulator 2 (SIRT2) in budding yeast. There are seven mammalian homologs of sirtuin (SIRT1–7), and SIRT1 is the closest homolog to SIRT2. SIRT1 modulates various key targets via deacetylation. In addition to histones, these targets include transcription factors, such as forkhead box O (FOXO), Ku70, p53, NF-κB, PPAR-gamma co-activator 1-alpha (PGC-1α), and peroxisome proliferator-activated receptor γ (PPARγ). SIRT1 has many biological functions, including aging, cell survival, differentiation, and metabolism. Genetic and physiological analyses in animal models have shown beneficial roles for SIRT1 in the brain during both development and adulthood. Evidence from *in vivo* and *in vitro* studies have revealed that SIRT1 regulates the cellular fate of neural progenitors, axon elongation, dendritic branching, synaptic plasticity, and endocrine function. In addition to its importance in physiological processes, SIRT1 has also been implicated in protection of neurons from degeneration in models of neurological diseases, such as traumatic brain injury and Alzheimer’s disease. In this review, we focus on the role of SIRT1 in the neuroendocrine system and neurodegenerative diseases. We also discuss the potential therapeutic implications of targeting the sirtuin pathway.

## Introduction

**FIGURE 1:**
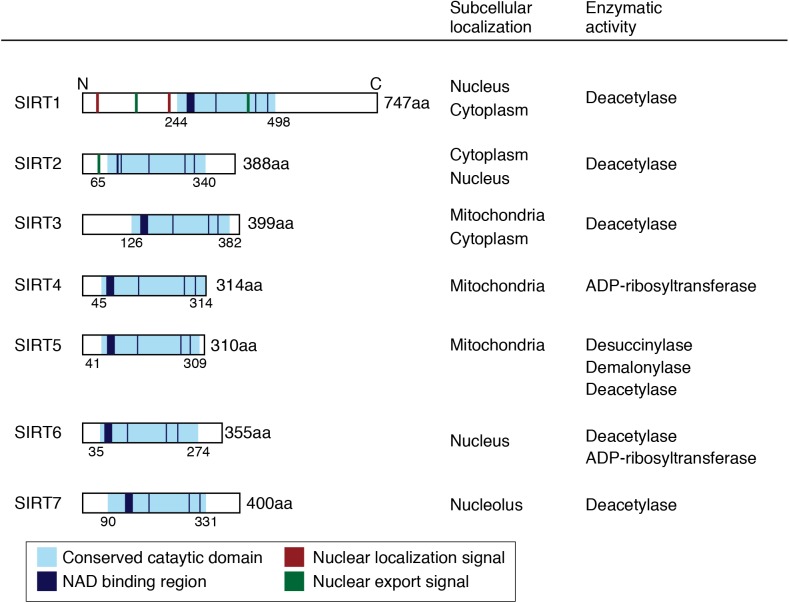
Schematic images and biological activities of human sirtuins. Conserved catalytic domains, NAD binding regions, nuclear localization signals, and nuclear export signals are shown in the schema.

The sirtuins are nicotinamide adenine dinucleotide (NAD)-dependent deacetylases, which are widely conserved proteins from bacteria to humans. The sirtuin protein was originally identified in *Saccharomyces cerevisiae* as silent information regulation 2 (SIRT2) (Klar et al., 1979; Rine et al., 1979), which regulates the lifespan by inhibiting genomic instability via chromatin modification. Sirtuins are categorized as class III histone deacetylases (HDACs). In mammals, seven sirtuin homologs (SIRT1–7) are categorized into four classes based on their DNA sequence. Sirtuins are typically composed of a conserved catalytic domain and variable N- and C-terminal domains. For example, the human *sirt1* gene is located on chromosome 10 and encodes a protein that is composed of 746 amino acids, which comprises the NAD-binding catalytic core domain. Sirtuins deacetylate histone lysine residues. This results in chromatin condensation, leading to transcriptional repression (Figure 1). However, several sirtuins do not appear to show

deacetylase activity. Silent information regulator 1 (SIRT1), SIRT2, SIRT3, and SIRT7 have NAD-dependent deacetylase activity; whereas SIRT4, SIRT5, and SIRT6 have weak or no detectable deacetylase activity. SIRT4 has adenosine diphosphate (ADP)-ribosyl transferase activity. SIRT5 shows more activity as an NAD-dependent demalonylase and desuccinylase than as a deacetylase. SIRT6 has both NAD-dependent deacetylase activity and ADP-ribosyl transferase activity (Haigis and Sinclair, 2010; Houtkooper et al., 2012). The crystal structure of the catalytic domain of human SIRT1 was identified, and revealed that SIRT1 activity is regulated by a C-terminal regulatory segment (Davenport et al., 2014). Intrinsically, disorder in the protein structure of SIRT1 might be related to its activity and physiological functions in the CNS (Khan and Lewis, 2005; Autiero et al., 2008; Sharma et al., 2012; Uversky, 2015).

Diversity in the subcellular localization of sirtuins can affect their cellular functions. SIRT1 is predominantly localized in the nucleus and deacetylates transcriptional factors, such as p53, FOXO, and NF-κB. It has been reported that SIRT1 shuttles into the cytoplasm during neuronal differentiation. SIRT2 is detected in the cytosol and colocalizes with microtubules and deacetylate α-tubulin. SIRT3, SIRT4, and SIRT5 are found in the mitochondria. SIRT3 is cleaved by the mitochondrial matrix processing peptidase (MPP) into a short form. The long form of SIRT3 can also localize in the nucleus. SIRT6 is associated with chromosome 19p13.3 in the nucleus. SIRT7 is a nuclear protein and regulates RNA polymerase 1-mediated transcription (Ford et al., 2006).

Silent information regulator 1, the most extensively studied mammalian ortholog of sirtuin, is classified as a class 1 sirtuin. Since the activity of SIRT1 depends on NAD+, the energy status of the cell and nutrient deprivation, such as fasting and caloric restriction, may affect its function (Rodgers et al., 2008). Although there have been some controversial aspects, SIRT1 can be associated with lifespan extension in many organisms. Accumulating studies suggest that SIRT1 plays vital roles in the development of the central nervous system (CNS) and brain functions. SIRT1 has been shown to mediate neuronal survival, neurite outgrowth, fate determination of neural precursor cells, and synaptic plasticity, through the deacetylation of target molecules (Guarente, 2011; Imai and Guarente, 2014). Lack of SIRT1 function impairs brain function, such as endocrine function, cognitive function, learning, and memory formation (Gao et al., 2010; Ramadori et al., 2010). Moreover, SIRT1 can ameliorate neurodegeneration in *in vivo* and *in vitro* models of Alzheimer’s disease, amyotrophic lateral sclerosis (ALS), and Wallerian degeneration (Araki et al., 2004; Qin et al., 2006b; Kim et al., 2007), suggesting that SIRT1 is important for neuronal protection against neurotoxic insults. Thus, the activation of SIRT1 may be a therapeutic target to overcome neurodegeneration, and several synthetic SIRT1 activators are attractive as putative drugs.

In this review, we summarize the role of sirtuins, especially SIRT1, in the CNS under physiological and pathological conditions. We also discuss the potential benefits of SIRT1 activators in the animal models of neurological diseases.

## Distribution of Sirt1 in the Central Nervous System

Silent information regulator 1 is ubiquitously expressed and demonstrates high expression in the brain (Sakamoto et al., 2004). SIRT1 is expressed in both neurons and glial cells (Chen J. et al., 2005; Hisahara et al., 2008; Cheng et al., 2014). Histological studies revealed that SIRT1 is prominently expressed in the hippocampus and hypothalamus within the adult mouse brain (Ramadori et al., 2008; Michan et al., 2010; Zakhary et al., 2010). The highest SIRT1 expression is observed in the early embryonic stage, and it gradually decreases during development. The expression levels of sirtuins seem to be affected by aging and pathological changes. SIRT1 deacetylation activity is downregulated in the aged brain and in several neurodegenerative models (Pallas et al., 2008; Quintas et al., 2012; Tang, 2017). In contrast, calorie restriction (CR) induces SIRT1 expression in the brain, as well as fat, kidneys, and liver (Cohen et al., 2004).

## SIRT1 Functions in Neurogenesis

Silent information regulator 1 is known to regulate pluripotency of embryonic stem cells and fate determination of neural progenitors. Mice carrying null alleles for SIRT1 show impaired embryogenesis (McBurney et al., 2003). The expression and/or acetylation levels of key pluripotency factors, such as Nanog, Oct-4, and Sox-2 are controlled by SIRT1 (Han et al., 2008; Yoon et al., 2014; Zhang et al., 2014). Overexpression of SIRT1 in neural tube of chick embryos decreases neurogenesis (Ichi et al., 2011). SIRT1 increases hairy and enhancer of split homolog-1 (Hes1) expression, which is important for neural stem cell maintenance, and decreases neurogenin2 (Neurog2) expression, which is involved in the promotion of neurogenesis. Treatment of nicotinamide, a SIRT1 inhibitor, enhances the differentiation of neural stem cells (Hu et al., 2014). Furthermore, nuclear translocation of SIRT1 regulates neuronal differentiation (Hisahara et al., 2008). SIRT1 predominantly shows cytoplasmic localization in neural precursor cells (NPCs), whereas its expression is mostly observed in the nucleus in differentiated NeuN-positive neurons. Nuclear SIRT1 interacts with nuclear receptor corepressor N-CoR, and this complex represses the transactivation of Hes1, leading to neuronal differentiation. Inhibition of SIRT1 using the pharmacological SIRT1 inhibitors (splitomicin or nicotinamide) or SIRT1-siRNA lentivirus decreases Tuj1-positive neurite length and neuronal differentiation both in culture and in the mouse brain after in utero electroporation (Hisahara et al., 2008). Furthermore, cytoplasmic SIRT1 promotes nerve growth factor (NGF)-induced neurite outgrowth in PC12 cells (Sugino et al., 2010). These observations suggest that subcellular localization of SIRT1 is essential for neuronal differentiation.

Silent information regulator 1 also modulates neurogenesis not only in the embryonic but also in adult rodent brain. Neurons are produced throughout life mainly in the germinal niches of the subventricular zone (SVZ) and dentate gyrus (DG) in the hippocampus. Reduced adult neurogenesis is known to be important for various brain functions, including learning and memory (Deng et al., 2010; Cameron and Glover, 2015). SIRT1 is expressed in proliferating cells in the SVZ and DG. Lentiviral-mediated knockdown of SIRT1 increases neurogenesis in the SVZ and hippocampus, whereas it does not affect the proliferation of neural precursors (Saharan et al., 2013). Genetic ablation of SIRT1 also promotes adult neurogenesis and activation of SIRT1 signaling by lentiviral-mediated forced expression or administration of resveratrol, which is known as a stimulator of SIRT1 activity, inhibits differentiation of adult neural precursors. These results suggest that SIRT1 is a negative regulator of adult neural precursor differentiation. However, other studies have demonstrated that stem cell-specific knockout of SIRT1 increases the proliferation and self-renewal rates of adult neural stem cells (Ma et al., 2014). Given these results, SIRT1 is likely to have dual functions in regulating both proliferation and differentiation of adult neural stem cells.

In addition, SIRT1 has been shown to enhance neurite and axon length as well as dendrite branching in hippocampal neurons. Upregulation of SIRT1 promotes axonogenesis and axon elongation via Akt deacetylation, which leads to inhibition of glycogen synthase kinase 3 (GSK3) activity (Li et al., 2013). Knockdown of SIRT1 enhances mTOR signaling and impairs neurite outgrowth and neuronal survival (Guo et al., 2011). Overexpression of SIRT1 or treatment with the SIRT1 activator resveratrol increases neuronal dendritic branching, possibly mediated by Rho-kinase (ROCK) activity (Codocedo et al., 2012). The tumor suppressor protein p53 is widely known to regulate neuronal apoptosis in pathological and physiological conditions. SIRT1 is a key regulator of p53 via deacetylation (Brooks and Gu, 2009; Revollo and Li, 2013). SIRT1 deacetylates p53 leading to the reduction of p53 activity and protecting cells from DNA damage (Luo et al., 2001; Vaziri et al., 2001). SIRT1 is associated with the maternally imprinted gene necdin and reduces p53-induced apoptosis in cortical neurons (Hasegawa and Yoshikawa, 2008). Thus, SIRT1 plays an important role during CNS development.

## SIRT1 Function in the Hypothalamus

**FIGURE 2:**
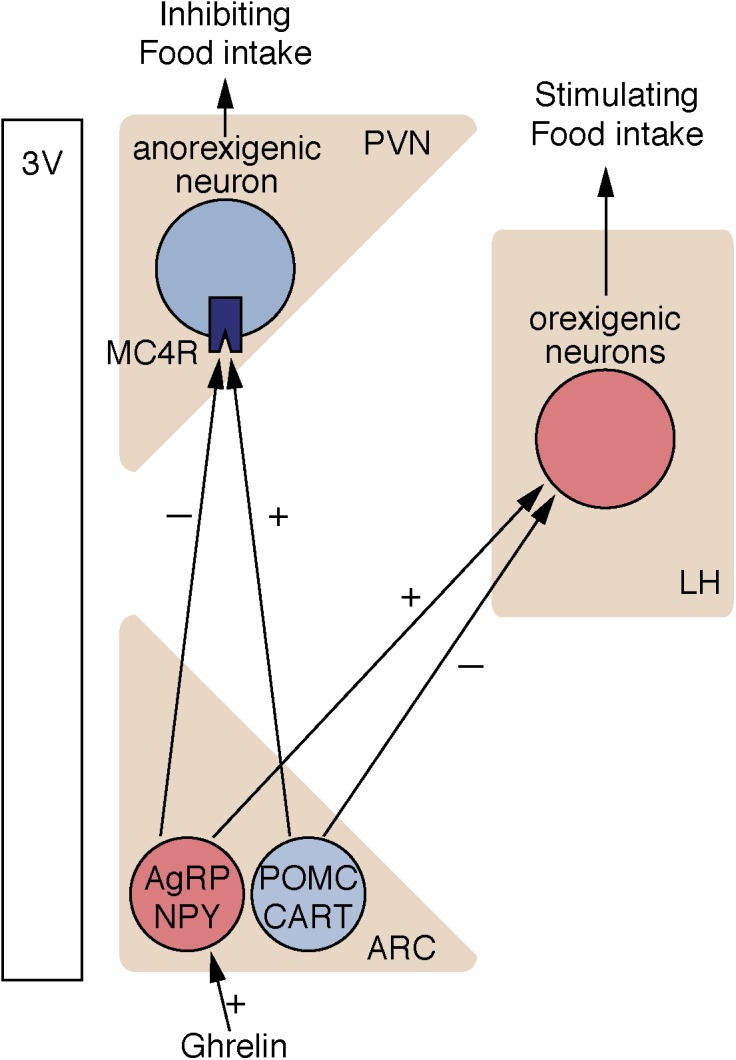
Neural circuits controlling food intake. Nutritional information is integrated into ARC in the hypothalamus. In ARC, orexigenic neurons express NPY, and AgRP, whereas anorexigenic neurons express POMC and CART. SIRT1 is expressed in both AgRP neurons and POMC neurons in the ARC. ARC, arcuate nucleus; PVN, paraventricular nucleus; LH, lateral hypothalamic area; AgRP, agouti-related peptide; NPY, neuropeptide Y; POMC, pro-opiomelanocortin; CART, cocaine- and amphetamine-related transcript; 3V, third ventricle.

Since sirtuins are NAD-dependent enzymes, they can be considered metabolic-sensor proteins (Coppari, 2012; Chang and Guarente, 2014; Satoh and Imai, 2014). The hypothalamus monitors endocrine responses and metabolic changes, which regulate food intake, the synthesis and secretion of hormones, and physiological rhythms. Hypothalamic nuclei, including the arcuate nucleus (ARC), ventromedial hypothalamic nucleus (VMH), paraventricular nucleus (PVN), and lateral hypothalamic area (LH) are involved in controlling food intake (Schwartz et al., 2000). There are two opposing types of neurons in the ARC: anorexigenic pro-opiomelanocortin (POMC) neurons, which secrete alpha-melanocyte stimulating hormone (α-MSH) and cocaine and amphetamine-regulated transcript peptide (CART); and orexigenic neuropeptide Y (NPY)/agouti-related peptide (AgRP) neurons, which produce NPY, AgRP, and gamma-aminobutyric acid (GABA). α-MSH secreted from POMC neurons activates melanocortin 3 and 4 receptors (MC3R and MC4R), promoting satiety. In contrast, AgRP inhibits these receptors and counteracts the function of α-MSH, thus promoting food intake (Dietrich et al., 2010; Figure 2).

Silent information regulator 1 is expressed in several hypothalamic nuclei, including anorexigenic POMC neurons and orexigenic AgRP neurons in the ARC, and other nuclei in the hypothalamus, such as LH and VMH. There have been conflicting reports regarding SIRT1 expression changes affected by fasting (Table 1). Compared to *ad libitum* feeding, fasting increased the ubiquitination of SIRT1 and decreased SIRT1 expression in the hypothalamus (Sasaki et al., 2010). Conversely, another study reported that fasting or diet restriction increased SIRT1 protein levels in the dorsomedial hypothalamus and LH (Satoh et al., 2010). Since SIRT1 expression changes (increased or decreased) are different in each tissue, tissue-specific analysis would be helpful. There are also varied findings on the role of hypothalamic SIRT1 on feeding behavior. Intracerebroventricular injection of Ex-527, a SIRT1 inhibitor, or small interfering RNA (siRNA)-mediated knockdown of SIRT1 in the ARC inhibits food intake due to the downregulation of AgRP and upregulation of POMC (Cakir et al., 2009). Specific knockout of SIRT1 in AgRP neurons decreases electrical responses of AgRP neurons to ghrelin, a stomach-derived peptide, and decreases ghrelin-induced feeding behavior (Dietrich et al., 2010). In contrast, it has been demonstrated that whole-body SIRT1 knockout mice are hyperphagic (Chen D. et al., 2005). Although specific knockout of SIRT1 in POMC neurons does not affect feeding behavior, mice lacking SIRT1 in POMC neurons demonstrate hypersensitivity to diet-induced obesity by reduced energy expenditure (Ramadori et al., 2010). In mice, adenovirus-mediated forced expression of SIRT1 in the mediobasal hypothalamus reduces food intake when compared to GFP-expressing control mice (Sasaki and Kitamura, 2010; Sasaki et al., 2010). Thus, SIRT1 is widely recognized as an important factor that controls food intake. More specific studies targeting individual neurons will reveal the detailed functions of hypothalamic SIRT1.

**Table 1:** The expression changes of SIRT1 in the hypothalamus.

The treatment that evoked the response (fasting/CR, duration)	SIRT1 expression changes (increase/decrease)	RNA or protein	Location of expression changes	Reference
Fasting in mice (Refeeding 24 h after 24 h starvation)	Increase	Protein	Liver	Rodgers et al., 2005, Nature
Fasting in mice (24 h starvation)	Increase	Protein	Brain, heart, muscle, white adipose, kidney	Kanfi et al., 2008, FEBS Letters
Fasting in mice (Refeeding 3 h after 24 h starvation)	Increase	Protein	Hypothalamus	Sasaki, 2010, Endocrinology
Fasting in mice (Refeeding 3 h after 24 h starvation)	Decrease	Protein	Cortex	Sasaki, 2010, Endocrinology
CR in rats (Lifelong restriction, starting immediately after weaning, with 60% of daily food)	Increase	Protein	Brain, fat kidney, liver	Cohen, 2004, Science
CR in mice (3–4 months old animals were subjected to a 30% CR diet)	Increase	Protein	Cortex, hippocampus	Chen et al., 2008, Exp Gerontology
CR in mice (3–4 months old animals were subjected to a 30% CR diet)	Decrease	Protein	Cerebellum, midbrain	Chen et al., 2008, Exp Gerontology
CR in mice (8–12 weeks old animals were subjected to a 60% of daily food for 14 days)	Increase	Protein	DMH, LH, SCN in the hypothalamus	Satoh, 2010, J Neurosci.


Agouti-related peptide neurons also regulate adaptive immune responses. Deletion of SIRT1 in AgRP neurons induces a pro-inflammatory state, which is associated with a decrease in regulatory T cell functions and consequent increase in effector T cell activity, leading to increased autoimmune disease susceptibility in mice (Matarese et al., 2013).

## SIRT1 and SIRT2 in Higher-Order Brain Functions

Sirtuins, especially SIRT1 and SIRT2, also mediate higher-order brain functions, such as learning, memory, and emotions (Donmez and Outeiro, 2013; Herskovits and Guarente, 2014). They regulate various neurological processes involving dendritic arborization, synaptic plasticity, and adult neurogenesis, which underlie these brain functions. Deletion of SIRT1 impairs cognitive functions. SIRT1 null mice exhibit deficits in short- and long-term associative memory and spatial learning (Michan et al., 2010). In SIRT1 null mice, there is less dendritic branching in the hippocampus, a key structure for learning and memory (Michan et al., 2010). Further, genes associated with synaptic function, membrane fusion, myelination, and amino acid and lipid metabolism were altered. Another study reported a direct role for SIRT1 in brain function (Gao et al., 2010). Deletion of SIRT1 in nestin-positive neural progenitor cells impaired memory and synaptic plasticity (Gao et al., 2010). SIRT1 conditional knockout mice have decreased memory performance in fear conditioning and novel object recognition tasks. In these mice, there is reduced expression of brain-derived neurotrophic factor (BDNF) and cAMP response binding protein (CREB), which are critical for synaptic plasticity and modulation of synapse formation, whereas microRNA (miRNA)-134 expression was upregulated. In the normal brain, SIRT1 cooperates with the transcriptional factor Yin Yang1 (YY1) and restricts the expression of miR-134 (Gao et al., 2010). SIRT1 deletion induces miRNA-134 expression, leading to the downregulation of BDNF and CREB, which subsequently impairs synaptic plasticity. Intraventricular injection of resveratrol, a natural compound that activates SIRT1, facilitates memory formation and synaptic plasticity in aged mice (Zhao et al., 2013). miR-124 and miR-134 expression decreased, whereas BDNF and CREB expression increased in resveratrol-treated mice. It has also been shown that miR-34c negatively regulates SIRT1 expression. Increased expression of miR-34c and decreased expression of SIRT1 were detected in mice with age-associated memory impairment and APPPS-21 mice, which are a model of amyloid pathology linked to Alzheimer’s disease (AD) (Yamakuchi et al., 2008; Zovoilis et al., 2011). These results suggest the possibility that decreased SIRT1 expression regulated by miRNAs may correlate with memory impairment. Furthermore, dysregulation of SIRT1 mediates obesity-induced memory impairments. High fat diet-induced obesity causes deficits in hippocampal-dependent spatial memory, synaptic plasticity, and altered gene expression. These effects were associated with decreased expression of SIRT1 (Heyward et al., 2012). Neuron-specific knockout of SIRT1 within the forebrain reversed obesity-induced hippocampal-dependent spatial memory deficits (Heyward et al., 2016). Meanwhile, another member of the HDAC family, HDAC2, negatively regulates synaptic plasticity and memory formation (Guan et al., 2009). Collectively, these findings suggest that targeting HDACs may be a key factor for regulating synaptic plasticity.

Silent information regulator 1 also modulates emotional responses, possibly associated with adaptive ability in a changing environment of food availability (Libert et al., 2011). Brain-specific SIRT1-knockout mice have less anxiety-like behaviors and more exploratory drive than their wild-type (WT) littermates. SIRT1 activates monoamine oxidase A (MAO-A) expression through the deacetylation of transcription factor nescient helix-loop-helix 2 (NHLH2), leading to a decrease of serotonin. MAO-A is the enzyme that degrades both serotonin and noradrenaline and is associated with mood disorders. Indeed, MAO inhibitors have been widely used for depression and several anxiety disorders (Preskorn, 2006). SIRT1 polymorphism frequencies were investigated in individuals with psychiatric disorders and controls; both rare and common alleles were associated with a higher risk of anxiety. Consistent with these observations, the mutations found in these individuals were linked to increased SIRT1 activity (Kishi et al., 2010; Jansen et al., 2016; Luo and Zhang, 2016). A genome-wide association study revealed that genetic variations near the *Sirt1* gene are significantly linked to major depressive disorder (Converge consortium, 2015). Later studies demonstrated decreased expression of SIRT1 in subjects with major depressive disorder compared to controls (Jansen et al., 2016; Luo and Zhang, 2016). An independent study of Japanese subjects demonstrated a significant association between SIRT1 SNP and major depressive disorders (Kishi et al., 2010). In a mouse model of depression, SIRT1 expression was increased in the nucleus accumbens (NAc), a brain region associated with reward and motivation. SIRT1 induction in the NAc promoted depression- and anxiety-like behaviors. Intra-NAc bilateral infusion of the SIRT1 agonist resveratrol in mice with viral-mediated overexpression of SIRT1 increased anxiety- and depression-like behavior in the open field, elevated plus maze, and forced swim tests. Intra-NAc infusion of the SIRT1 antagonist EX-527, or viral-mediated knockdown of SIRT1 in the NAc reduced those behavioral effects. Hippocampal SIRT1 is associated with chronic stress-induced depressive behavior (Abe-Higuchi et al., 2016). Chronic stress reduces SIRT1 expression in the hippocampus, and decreases dendrite length and spine density. Depressive behaviors in the mice subjected to chronic stress were reversed by viral-mediated overexpression of SIRT1 into the DG area of hippocampus (Abe-Higuchi et al., 2016).

Furthermore, sirtuins control behavioral responses to cocaine and morphine in the NAc. Addictive drugs, such as cocaine, induce gene expression changes in the NAc, which affect reward circuitry (Freeman et al., 2001; McClung and Nestler, 2003; Yao et al., 2004; Hyman et al., 2006). It is known that increased acetylation of histone H3 or H4 causes transcriptional activation, whereas increased methylation of histone H3 at Lys9 causes transcriptional repression. Chromatin immunoprecipitation (ChIP) analysis revealed that chronic administration of cocaine increases or decreases histone acetylation in the NAc at the gene promoters encoding the genes known to show drug-induced upregulation or downregulation, respectively (Kumar et al., 2005; Renthal et al., 2008). In a subsequent study, genome-wide ChIP followed by promoter microarray analysis (ChIP-chip) methods demonstrated that SIRT1 and SIRT2 were the targets of increased histone H3 acetylation in the NAc after chronic cocaine administration. Furthermore, the catalytic activity of these sirtuins was increased in the NAc by chronic cocaine administration (Renthal et al., 2009). Overexpression of SIRT1 or SIRT2 in the NAc using adeno-associated viruses (AAV) promotes the rewarding effects of both cocaine and morphine reward, while genetic deletion of SIRT1 in the NAc decreases drug reward (Ferguson et al., 2013).

Collectively, dysregulation of sirtuins are involved in diverse phenomena associated with higher brain dysfunction, including synaptic dysfunction, altered neurotransmitter secretion, and genetic variations.

## Sirtuins and Neurodegenerative Disorders

Numerous studies have demonstrated that sirtuins mediate a variety of neurodegenerative disorders (Gan and Mucke, 2008; Donmez, 2012; Herskovits and Guarente, 2014). Altered sirtuin expression and/or activation might be associated with disease development and progression (Table 2). Beneficial effects of SIRT1 activation using genetic manipulation and pharmacological treatment have been reported in various animal models for neurodegenerative diseases (Table 3). In contrast, inhibition of SIRT1 signaling has also been shown to exert neuroprotective effects. Other sirtuins, especially SIRT2, are reported to be involved in neurodegenerative disorders. Thus, the sirtuin family shows diverse effects. The following sections discuss the pleiotropic effects of sirtuins in neurodegenerative disorders.

**Table 2:** The expression changes of sirtuins in neurological diseases and animal models.

Sirtuins	Sirtuin expression changes (increase/decrease)	RNA or protein	Location of expression changes	Human or mouse	Disease or animal model	Reference
SIRT2	Decrease	Protein	Cultured cerebellar granule cells from Wld^s^ mice	Mouse	Wld^s^	Suzuki and Koike, 2007
SIRT1	Decrease	Protein	Spinal cord	Mouse	SCI	Chen, 2017
SIRT1	Increase	Protein	Injured-side cortex	Mouse	TBI	Zhao, 2012
SIRT1	Increase	Protein	Cortex	Rat	SAH	Zhang, 2016
SIRT1	Increase	Protein	Peri-infarct regions of injured-side cortex	Mouse	MCAO	Hernandez-Jimenez, 2013
SIRT1	Increase	Protein	Acute and chronic active lesion in MS brain CD4+, CD68+, GFAP+, oligodendrocytes	Human	MS	Tegla, 2013
SIRT1	Increase	Protein	GFAP+ cells in typical inflammatory perivascular cuffs in brain	Mouse	EAE	Prozorovski, 2008
SIRT1	Decrease	Protein, mRNA	Parietal cortex	Human	AD	Julien, 2009
SIRT3	Decrease	Protein	Frontal cortex	Human	AD	Lee, 2018
SIRT6	Decrease	Protein	Temporal cortex	Human	AD	Kaluski. 2017
SIRT1	Increase	Protein	Forebrain	Mouse	AD	Kim, 2007
SIRT1	Decrease	Protein	Frontal cortex	Human	PD	Singh, 2017
SIRT1 (80 kDa)	Increase	Protein	Temporal cortex	Human	PD	Singh, 2017
SIRT1	Increase	Protein	Spinal cord	SOD1G37R mouse	ALS (severe neurodegeneration)	Kim, 2007
SIRT3	Decrease	mRNA	Spinal cord, brain stem	SOD1 G93A mouse	ALS (end stage)	Buck, 2017


**Table 3:** The role of SIRT1 in animal models of neurodegenerative diseases.

Manipulations of SIRT1	Effects on CNS	Human or mouse	Animal model	Reference
Resveratrol	Decreased axonal degeneration	Mouse	Wallerian degeneration (DRG explant culture)	Araki et al., 2004
siRNA-mediated knockdown of SIRT1 or Sirtinol	Decreased NAD-dependent axonal protection	Mouse	Wallerian degeneration (DRG explant culture)	Araki et al., 2004
SRT1720	Improved locomotor recovery, decreased proinflammatory cytokine expression, decreased accumulation of macrophages/microglia	Mouse	SCI	Chen et al., 2017
Resveratrol	Improved motor functional recovery, decreased motor neuron loss	Rat	SCI	Zhao et al., 2017
siRNA-mediated knockdown of SIRT1 or SIRT1 inhibitor	Increased apoptosi in cultured cortical neurons, increased ERK1/2 activation after TBI	Mouse	TBI (Primary cortical neuron culture)	Zhao et al., 2012
Resveratrol	Induced ischemic tolerance	Mouse	Transient MCAO	Koronowski et al., 2017
Homozygous deletion of SIRT1	Increased infarct volume	Mouse	Permanent MCAO	Hernandez-Jimenez et al., 2013; Liu et al., 2013
SIRT1 activator A3	Decreased infarct volume	Mouse	Permanent MCAO	Hernandez-Jimenez et al., 2013
Nicotinamide	Decreased infarct volume	Mouse	Permanent MCAO	Liu et al., 2009
Resveratrol	Increased survival of retinal ganglion cells	Mouse	EAE induced by PLP immunization of SJL/J mouse	Shindler et al., 2007
SIRT1-overexpressing mouse (pCaMKIIα-tTA; pTRE-SIRT1/mito/eYFP)	Decreased EAE clinical symptoms, reduced demyelination and axonal injury	Mouse	EAE induced by MOG immunization of C57BL/6 mouse	Nimmagadda et al., 2013
Virus-mediated expression of SIRT1	Decreased Aβ peptide in primary Tg2576 neurons	Mouse	AD (Primary cortical neuron culture of Tg2576 mouse)	Qin et al., 2006, JBC
Virus-mediated expression of SIRT1	Increased survival neurons in CA1	Mouse	AD (Inducible p25 transgenic mouse)	Kim, 2007
Resveratrol	Decreased thioflavine S-positive plaques in cortex, striatum, and hypothalamus	Mouse	AD (Tg19959 mouse)	Karuppagounder et al., 2009
Resveratrol	Decreased Aβ peptide in primary neurons	Mouse	AD (Primary cortical neuron culture of J20 APP mice)	Vingtdeux et al., 2010
Resveratrol	Decreased neurodegeneration	Mouse	PD (MPTP treatment)	Mudo et al., 2012
SIRTl-overexpressing mouse (NSE-SIRT1 mice)	TH-positive neurons	Mouse	PD (MPTP treatment)	Kakefuda et al., 2009
Resveratrol	Extended lifespan, delayed onset of symptoms, increased survival of motor neurons	Mouse	ALS (SOD1G93A mouse)	Han et al., 2012; Mancuso et al., 2014
SIRT1-overexpressing mice (PrP-SIRT1 mice)	Extended lifespan	Mouse	ALS (SOD1G93A mouse)	Watanabe et al., 2014


### Wallerian Degeneration

There are numerous studies reporting the protective effects of SIRT1 against axonal degenerative processes. In this section, we introduce early studies, which suggest that activation of the SIRT1 pathway possibly delayed Wallerian degeneration. Wallerian degeneration is the anterograde degeneration of axons and synapses after injury (Coleman, 2005). This process usually occurs about 1.5 days after injury. However, Wallerian degeneration mutant (Wld^s^) mice, which carry an autosomal dominant mutation in chromosome 4, show delayed Wallerian degeneration for 2–3 weeks (Lunn et al., 1989; Coleman and Freeman, 2010). The Wld^s^ mutation is an 85-kb tandem triplication, which causes overexpression of chimeric Wld^s^ protein. The mutation region comprises two associated genes: an E4-type ubiquitin ligase Ube4b (or Ufd2a), and the protein associated with the NAD salvage pathway in mammals, nicotinamide mononucleotide adenylyltransferase 1 (Nmnat1). It has been suggested that Nmnat1 activity is responsible for the protective effect of Wld^s^ protein thorough SIRT1 (Araki et al., 2004). Knockdown of SIRT1 or treatment with the SIRT2 inhibitor, sirtinol, inhibits NAD-dependent axonal protection in cultured dorsal root ganglion neurons, which had induced degeneration by the removal of cell bodies. Meanwhile, Nmnat1/NAD-induced neuroprotective effects independent of SIRT1 have also been demonstrated. In addition to Nmnat1, the N-terminal of Wld^s^ is also required for axonal protection mediated by Wld^s^ (Avery et al., 2009). The extranuclear translocation and axon localization of NMNAT1 protein may exert neuroprotective potency *in vivo* (Babetto et al., 2010). Transgenic mice carrying axon-targeted Nmnat1 showed robust axonal protection after axotomy. Further, SIRT2 can also modulate resistance to axonal degeneration. Tubulin acetylation is associated with microtubule stability. Increased microtubule acetylation was observed in cultured cerebellar granule cells from Wld^s^ mice (Suzuki and Koike, 2007). SIRT2 is an NAD-dependent tubulin deacetylase, and SIRT2 expression was decreased in these cells. Overexpression of SIRT2 abolished microtubule hyperacetylation and resistance to axonal degeneration in the cells of Wld^s^ mice, whereas lentiviral-mediated knockdown of SIRT2 enhanced microtubule acetylation and resistance to degeneration in wild-type cerebellar granule cells (Suzuki and Koike, 2007).

### Spinal Cord Injury (SCI)

Although the direct effect of SIRT1 on axonal regeneration remains obscure, activation of SIRT1 has been shown to exert beneficial effects on motor function in the animal model of spinal cord injury (SCI). In SCI, following the initial damage to the spinal cord, tissues are directly damaged and disrupted. Injured neurons, glia, and vasculature cause oxidative stress, free radical generation, edema, and further inflammatory reactions which induce secondary injury, leading to the expansion of spinal cord damage and sustained impairment of neurological function (Ahuja et al., 2017). Immune responses after SCI, such as infiltration and activation of inflammatory cells and inflammatory cytokine production, mediate the pathogenesis of SCI. Overwhelming immune responses aggravate the injury. In this regard, controlling immune responses contributes to functional recovery after SCI (Courtine et al., 2011; Donnelly et al., 2011). SIRT1 expression at the lesion site, where inflammatory responses must be evident was decreased 4 h after SCI and persisted for 3 days. Treatment with an SIRT1 activator, SRT1720, reduced inflammatory cytokines and inflammatory cells and promoted functional recovery after SCI (Chen et al., 2017). Mx1-Cre-mediated knockout of SIRT1 in mice, which ablated SIRT1 expression in inflammatory cells, such as macrophages, neutrophils, dendritic cells, T cells, and B cells, caused increased levels of inflammatory cytokines and severe inhibition of motor recovery compared to those in wild-type mice. These findings suggest that the anti-inflammatory role of SIRT1 contributes to beneficial effects on motor function after SCI. Consistent with these observations, treating rats with resveratrol had neuroprotective effects after SCI through the enhancement of autophagy and inhibition of apoptosis regulated by SIRT1/AMP-activated protein kinase (AMPK) signaling (Zhao et al., 2017). Resveratrol also promoted motor function recovery after SCI. Thus, in addition to its neuroprotective functions, the anti-inflammatory effects of SIRT1 may also promote motor function recovery after SCI.

### Traumatic Brain Injury

Silent information regulator 1 was shown to prevent neuronal apoptosis in a transection model *in vitro* and weight-drop model *in vivo*, which mimics traumatic brain injury (TBI). SIRT1 expression was increased 30 min to 24 h after TBI with a peak level at 6 h after injury in the animal model of TBI. Inhibiting SIRT1 using a pharmacological inhibitor (salermide) or siRNA decreased ERK1/2 activation and enhanced neuronal apoptosis after mechanical traumatic injury *in vivo* (Zhao et al., 2012). Inhibition of ERK reduced apoptosis and decreased SIRT1 upregulation after TBI. ERK1/2 activation has neuroprotective functions and mediates the beneficial effects of neuroprotectants (Fukunaga and Miyamoto, 1998; Guerra et al., 2004; Karmarkar et al., 2011). However, other studies have demonstrated that ERK1/2 activation can have neurotoxic signals *in vitro* and *in vivo* (Alessandrini et al., 1999; Stanciu et al., 2000; Lesuisse and Martin, 2002). In the adult mouse brain, SIRT1 is predominantly expressed in the cytosol, where ERK1/2 is localized (Tanno et al., 2007; Li et al., 2008). Considering these observations, there may be a synergistic relationship between SIRT1 and ERK pathways that regulate neuronal apoptosis following TBI. Furthermore, it has been reported that upregulation of SIRT1 is involved in the neuroprotective effects induced by natural components, such as vitamin E and omega-3 fatty acids in a TBI model (Wu et al., 2006, 2007; Aiguo et al., 2010).

### Stroke

Stroke is a cerebrovascular disease that can lead to death or neuronal dysfunction. Vascular occlusion causes a deprivation of oxygen and energy, followed by the collapse of ionic gradients across the cell membrane and neuronal death caused by an excess release of excitatory neurotransmitters. These sequential events result in the formation of reactive oxygen species, gene expression changes, and induction of inflammatory processes, which contribute to irreversible tissue damage (Iadecola and Anrather, 2011). A variety of animal stroke models have been widely used. The transient or permanent middle cerebral artery occlusion (MCAO) model is a well-established model for human ischemic stroke (Koizumi et al., 1986; Longa et al., 1989; Hossmann, 1998). Oxygen and glucose deprivation (OGD) is a useful *in vitro* model alternative to the animal ischemia model (Ogawa et al., 1990). SIRT1 expression is decreased after transient MCAO or prolonged OGD (Yan et al., 2013), whereas its expression is not altered after moderate ischemia and short OGD (Wang et al., 2013). Since both beneficial and detrimental effects of SIRT1 have been reported, the protective effect of SIRT1 in ischemia remains controversial. Initial studies reported that treatment with resveratrol shows neuroprotective effects in OGD in organotypic hippocampal slices and global cerebral ischemia in rats (Raval et al., 2006, 2008). A subsequent study showed that both ischemic preconditioning, which develops brain tolerance to a secondary ischemic damage, and resveratrol treatment had neuroprotective effects, possibly via the downregulation of mitochondrial uncoupling protein 2 (UCP2) (Della-Morte et al., 2009). UCP-2 reduces mitochondrial membrane potential and inhibits ATP production. SIRT1 binds to the UCP-2 promoter and regulates its transcription. Since the SIRT1 inhibitor sirtinol reversed the effect of resveratrol, SIRT1 activity seems to be required for resveratrol-mediated neuroprotective effects in cerebral ischemia. Emerging studies demonstrated that the glycolytic function of SIRT1 mediates resveratrol-induced ischemic tolerance in an animal model of stroke (Koronowski et al., 2017). Neuron-specific knockout of SIRT1 in the adult brain abolishes resveratrol-induced neuroprotection in MCAO. Altered glucose metabolism and impaired glycolytic ATP production were observed in neuron-specific SIRT1-knockout mice. The neuronal activities of SIRT1 seem to precede resveratrol-induced neuroprotection.

Ischemic brain injury depletes intracellular NAD^+^, which is indispensable for the catalytic activity of SIRT1 (Endres et al., 1997). Treatment with NAD ameliorates ischemic injury in a rat model of transient focal ischemia (Ying, 2007; Ying et al., 2007). Moreover, nicotinamide phosphoribosyltransferase (Nampt), which is the rate-limiting enzyme in NAD+ production within the NAD+ salvage pathway, mimics the positive effects of NAD+ against stroke (Rongvaux et al., 2003; Revollo et al., 2007; Yang et al., 2007). Overexpression of Nampt reduces ischemic infarct in experimental cerebral ischemia rats, and SIRT1 global knockout blocks this effect (Wang et al., 2011). LKB1 deacetylation and AMPK activation regulated by SIRT1 contribute to the neuroprotection of Nampt. The results from *in vitro* and *in vivo* studies demonstrate that increased autophagy through SIRT1-dependent TSC2-mTOR-S6K1 signaling is also involved in Nampt-induced neuroprotection in cerebral ischemia (Wang et al., 2012). Furthermore, SIRT1-knockout mice demonstrate greater infarct volumes after MCAO than their wild-type counterparts (Hernandez-Jimenez et al., 2013; Liu et al., 2013). Pharmacological modulation using the SIRT1 activator A3 decreases the infarct volume, while the SIRT1 inhibitor sirtinol increases the infarct volume in the MCAO model. Increased acetylation of p53 and NFκB via inhibition of SIRT1 aggravate ischemic injury.

Neurovascular protection of SIRT1 was also reported in cerebral hypoperfusion induced by bilateral common carotid artery stenosis (Hattori et al., 2014). SIRT1-overexpressing transgenic mice preserved cerebral blood flow after cerebral hypoperfusion. SIRT1 is implicated in the neuroprotective effects of experimental subarachnoid hemorrhage (SAH) in rats (Zhang et al., 2016). SIRT1 expression was increased and peaked 24 h after SAH. Pharmacological inhibition of SIRT1 using sirtinol exacerbates neuroinflammation and neuronal apoptosis after SAH, whereas activation of SIRT1 using A3 reduces SAH-induced early brain injury.

Silent information regulator 1 activation does not always exert neuroprotective functions (Ng and Tang, 2013; Sansone et al., 2013). Overexpression of human SIRT1 in neurons under the control of the neuron-specific enolase promoter does not induce neuroprotection in mice (Kakefuda et al., 2009). An NAD+ precursor and a SIRT1 inhibitor, nicotinamide, reduces infarct size in a permanent focal cerebral ischemic model (Liu et al., 2009). Other sirtuins are also involved in ischemic processes. Mitochondrial NAD + -dependent SIRT3 mediates the beneficial effects of ketone bodies after MCAO (Yin et al., 2015). SIRT6 overexpression decreases cerebral infarction and attenuates neurological deficits after MCAO/reperfusion (Zhang et al., 2017). SIRT6 activates nuclear factor-erythroid 2-related factor-2 (NRF2), which is a basic leucine zipper transcription factor that regulates the expression of antioxidant proteins. NRF2-knockout mice abolish the neuroprotective effects of SIRT6. As such, the activation of NRF2 induced by SIRT6 overexpression may be implicated in its protective effects after stroke.

### Multiple Sclerosis (MS)

Multiple sclerosis (MS) is a chronic inflammatory demyelinating disease of the CNS, mostly driven by autoimmune causes. Immune cells infiltrate the CNS and attack myelin sheaths, leading to demyelination, axonal damage, and neurological disability (Hauser and Oksenberg, 2006; Trapp and Nave, 2008). CD4+ T cells are critical effector cells in CNS inflammation (Goverman, 2009). Individuals with MS often show permanent axonal damage and neuronal loss. MS lesions are often located in the brain, spinal cord, cranial nerves, and optic nerve. Experimental autoimmune encephalomyelitis (EAE) is one of the best available models for human MS. Several studies have demonstrated that pharmacological activation of SIRT1 confers protective functions in mouse models of MS. Intravitreal injection of a SIRT1 activator, SRT501 (resveratrol) or SRT647, attenuates retinal ganglion cell death in optic neuritis of a relapsing-remitting EAE model in SJL mice (Shindler et al., 2007). This neuroprotective effect is blocked by the SIRT1 inhibitor, sirtinol. A subsequent study demonstrated that treatment with SRT501 preserves axonal density in the spinal cord when compared to vehicle treatment (Shindler et al., 2010). Similar beneficial effects were also reported in chronic EAE in C57BL/6 mice and in a virus-induced CNS demyelination model (Fonseca-Kelly et al., 2012; Khan et al., 2014). Genetic overexpression of SIRT1 in EAE mice support the beneficial effects of SIRT1. Transgenic mice with neuron-specific overexpression of SIRT1 were induced with chronic EAE by immunization with myelin oligodendrocyte glycoprotein (MOG) peptide, and showed suppressed EAE clinical symptoms when compared to wild-type EAE mice (Nimmagadda et al., 2013). Increased BDNF and NAD could be responsible for the neuroprotective effects observed in this mouse line.

In contrast, other studies have shown that inhibition of SIRT1 contributes to the amelioration of EAE. Adult neural progenitor cells could be a possible regenerative tool to compensate for neuronal loss after CNS damage. However, most NPCs generate more glial cells than neurons, and the compensation for damaged neurons is insufficient (Ridet et al., 1997). SIRT1 expression was increased in GFAP-positive cells around EAE inflammatory lesions. Mild oxidation induced by buthionine sulfoximine or diethyldithiocarbamate, or resveratrol-induced SIRT1 activation, suppressed proliferation of NPCs and increased differentiation toward astrocytes, whereas redox conditions induced by lipoic acid of N-acetylcysteine showed opposite effects (Prozorovski et al., 2008). Upregulation of SIRT1 in oxidative conditions promotes SIRT1 binding to Hes1 and inhibits Mash1, resulting in NPC differentiation toward astrocytes. NPC-specific knockout of SIRT1 also increases the generation of oligodendrocyte progenitor cells (OPCs), which are the origin of the myelin-forming glial cells, oligodendrocytes (Rafalski et al., 2013). SIRT1 inactivation enhances remyelination and delays onset of paralysis in a chronic EAE model. Furthermore, global knockout of SIRT1 inhibits production of pro-inflammatory T helper 17 (Th17) cells and ameliorates EAE clinical scores in Th17 cell-mediated autoimmune diseases (Lim et al., 2015). Pharmacological inhibition of SIRT1 using Ex-527 also attenuates the infiltration of immune cells into the spinal cord and ameliorates EAE. Th17 cells are involved in the onset and pathogenesis of autoimmune diseases. The transcription factor, RAR-related orphan receptor γ-t (RORγt), regulates Th17 cell differentiation (Ivanov et al., 2006; Kebir et al., 2007). SIRT1 physically interacts with RORγt and promotes Th17 differentiation via deacetylation of RORγt. Therefore, SIRT1 inhibition can exert both beneficial and detrimental effects on EAE. Although specific deletion in particular cells may be challenging, distinctive pharmacological inhibition of SIRT1 in NPC and/or immune cells may serve as a potential treatment for MS.

#### Studies in Individuals With MS

Studies on the brains of individuals with MS revealed increased SIRT1 expression in acute and chronic lesion sites, whereas its expression is rarely detected in normal brains (Tegla et al., 2014). A high level of SIRT1 expression is observed in MS plaques. Moreover, CD4+ and CD68+ inflammatory cells, oligodendrocytes, and glial fibrillary acidic protein (GFAP)-positive astrocytes in MS plaques co-localize with SIRT1. Furthermore, SIRT1 expression in peripheral blood mononuclear cells (PBMCs) obtained from patients with MS that had relapses was decreased compared to that in controls and stable patients with MS. Responders to glatiramer acetate treatment in relapsing-remitting MS show higher SIRT1 expression (Hewes et al., 2017). These results suggest that low levels of SIRT1 can be used as a putative biomarker for MS patients.

### Alzheimer’s Disease

Deposition of aggregate amyloid-β (Aβ), as well as tau phosphorylation and neurofibrillary tangles, are well-characterized hallmarks of AD. These abnormal protein aggregations are considered to be related to neurodegeneration that causes neuronal death, brain atrophy, and subsequent memory loss and cognitive deficits in AD (Hardy and Selkoe, 2002; Ballard et al., 2011). Many studies have demonstrated that SIRT1 activation has beneficial effects in diverse animal models of AD, and activation of SIRT1 has therapeutic potential for AD (Bonda et al., 2011). Numerous systematic reviews discussing the beneficial functions of SIRT1 in AD have been published (Bonda et al., 2011; Donmez and Outeiro, 2013; Herskovits and Guarente, 2014; Ng et al., 2015); we therefore focus on recent findings on the role of SIRT1 and other sirtuins in AD in this section.

Silent information regulator 1 activation using resveratrol and SIRT1 overexpression has been shown to reduce amyloid plaque formation and confer protective effects in diverse animal models of AD (Chen J. et al., 2005; Qin et al., 2006b; Kim et al., 2007; Karuppagounder et al., 2009; Vingtdeux et al., 2010). CR, which is known to induce SIRT1 activation, attenuates amyloid toxicity both in murine and primate AD models (Wang et al., 2005; Qin et al., 2006a). SIRT1 has also been shown to reduce neurofibrillary tau pathology (Green et al., 2008; Min et al., 2010). Furthermore, the role of SIRT1 appears to be involved in the association between neuronal energy metabolism and AD. Increased accumulation of sterol was observed in the brains of humans with AD and rodent models of AD. Prevention of the accumulation of ceramides and cholesterol resulted in neuronal protection from cell death induced by Aβ. These findings suggest that perturbed cholesterol metabolism could be responsible for triggering neurodegenerative cascades in AD (Cutler et al., 2004; Xiong et al., 2008; Bandaru et al., 2009; Fernandez et al., 2009; Panchal et al., 2010). SIRT1 and AMPK have been shown to positively regulate each other’s activities and mediate various processes, such as cellular metabolism, mitochondrial function, and inflammation (Ruderman et al., 2010). The plant-derived protein osmotin has been shown to ameliorate Aβ-induced synaptic dysfunction and memory impairment in rats (Shah et al., 2014; Teller et al., 2015). Subsequent studies demonstrated that osmotin treatment reduces cholesterol biosynthesis and exerts beneficial effects through the activation of the SIRT1/AMPK pathway in an AD mouse model (Shah et al., 2017a,b). The drugs that inhibit cholesterol biogenesis and/or activation of SIRT1/AMPK axis may serve as candidates for developing therapies against excess cholesterol accumulation in AD.

Possible roles for other sirtuins in AD have been reported. Recent studies revealed that apolipoprotein E4 is a genetic factor in late-onset AD. SIRT3 expression is decreased in the frontal cortex of patients with AD, and dysregulation of SIRT3 induces p53-mediated mitochondrial and neuronal damage in AD (Lee et al., 2018). In addition, individuals with AD show decreased expression of SIRT6 (Kaluski et al., 2017). SIRT6 regulates DNA repair and maintenance of genomic stability via the base excision repair pathway (Giblin et al., 2014; Kugel and Mostoslavsky, 2014). SIRT6-deficient mice show genomic instability, progeroid features, and severe metabolic deficits, such as fatal hypoglycemia (Xiao et al., 2010; Etchegaray et al., 2013; Jung et al., 2016). Brain-specific SIRT6-knockout mice show increased DNA damage, apoptosis, and learning impairments (Kaluski et al., 2017). Lack of SIRT6 induces tau protein stabilization and increases tau phosphorylation via activation of glycogen synthase kinase 3 (GSK3). Individuals with AD show a reduction in SIRT6 expression. Notably, there is a further reduction with increased severity of Braak stages. These findings suggest that SIRT6 is required to keep the brain healthy by preventing naturally occurring DNA damage.

### Parkinson’s Disease

Recently, accumulating studies have revealed the relationship between sirtuins and Parkinson’s disease (PD) *in vitro* and *in vivo*. PD is an age-associated neurodegenerative disease characterized by motor disorders due to the degeneration and dysfunction of dopaminergic neurons in the substantia nigra and striatum. Mitochondrial abnormalities and Lewy bodies, mainly containing misfolded and aggregated α-synuclein protein, are implicated in the pathology of PD. SIRT1 activity is reduced in post-mortem brain tissue obtained from individuals with PD (Singh et al., 2017). SIRT1 activity is also decreased in induced pluripotent stem cell (iPSC)-derived dopaminergic neurons carrying a glycine to serine mutation (G2019S) in leucine-rich repeat kinase 2 (LRRK2), which is causally associated with PD and is involved in the impairment of mitochondrial function (Schwab et al., 2017). Three heterozygous sequence variants within the promoter regions of *SIRT1* gene in patients with sporadic PD were identified, but these were absent in controls. These variants are associated with the reduced expression of SIRT1 in PD patients (Zhang et al., 2012). Several studies have proposed mechanisms for the protective effects of SIRT1 on PD. The 1-methyl-4-phenyl-1,2,3,6-tetrahydropyridine (MPTP)-treated animal model is widely used as an animal model of PD. Administration of resveratrol or genetic overexpression of peroxisome proliferator-activated receptor-gamma coactivator-1α (PGC1α), a transcriptional coactivator that is deacetylated by SIRT1, decreases MPTP-induced neuronal degeneration (Mudo et al., 2012). Cellular models of PD have implicated SIRT1-mediated autophagy and mitophagy under α-synuclein-induced toxicity (Sampaio-Marques et al., 2012). Knockout of SIRT1 worsened movement in an MPTP-treated model, whereas other studies reported that SIRT1 did not prevent neuronal damage to tyrosine hydroxylase (TH)-positive dopaminergic neurons induced by MPTP (Kakefuda et al., 2009; Zhang et al., 2018). Thus, the agents inducing SIRT1 activation and/or expression could be therapeutic drugs for PD. However, a few studies suggested that SIRT1 inhibition does show neuroprotective effects. 1-methyl-4-phenylpyridinium (MPP+) is the active metabolite of MPTP, and has dopaminergic toxicity (Dauer and Przedborski, 2003). Knockdown of SIRT1 using siRNA reduces MPP + -induced apoptosis in SH-SY5Y human neuroblastoma cells (Park et al., 2011). Therefore, the beneficial effects of SIRT1 activating compounds on PD model may need to be assessed in various conditions.

Silent information regulation 2 inhibition contributes to reduced α-synuclein toxicity. Pharmacological inhibition or siRNA-mediated inhibition of SIRT2 decreases the number of α-synuclein inclusions in a cellular model of PD (Outeiro et al., 2007). SIRT2 deacetylates α-synuclein, and knockdown of SIRT2 suppresses α-synuclein aggregation and toxicity in a mouse model of PD (de Oliveira et al., 2017). The acetylation of α-synuclein promotes the clearance of α-synuclein inclusions via autophagy and exerts neuroprotective effects in cultured neurons, whereas blocking the acetylation of α-synuclein causes the loss of nigral dopaminergic neurons. Other studies showed that deletion of SIRT2 reduces MPTP-induced neuronal damage in TH-positive cells through increased acetylation of FOXO3a and reduces expression of a proapoptotic factor, Bim, thus blocking the apoptotic pathway (Liu et al., 2012). These results suggest that SIRT2 inhibition may have beneficial effects for PD.

### Amyotrophic Lateral Sclerosis (ALS)

Amyotrophic lateral sclerosis is a progressive neurodegenerative disease that affects motor neurons in the brain and spinal cord. ALS is a uniformly fatal disorder and causes death about 2–5 years after onset, mostly because of respiratory paralysis. Mutations in various genes have been identified in ALS, including superoxide dismutase (SOD1) (Rosen et al., 1993) and TAR DNA-binding protein 43 (TDP43) (Arai et al., 2006; Neumann et al., 2006; Kabashi et al., 2008; Sreedharan et al., 2008; Van Deerlin et al., 2008). It has been reported that sirtuin expression is altered in both mouse models of as well as patients with ALS (Kim et al., 2007; Lee et al., 2012; Korner et al., 2013; Buck et al., 2017). Treating with resveratrol or lentivirus-mediated forced expression of SIRT1 protects against neurodegeneration in the SOD1 mutant mouse model of ALS (Kim et al., 2007; Han et al., 2012; Mancuso et al., 2014). SOD mutant mice that show consistent pan-neural expression of exogenous SIRT1 have extended lifespans compared to those without this expression (Watanabe et al., 2014).

SIRT3 has also received attention for its role in ALS. Expression of SIRT3 decreases in the spinal cord and brain stem during the progression of diseases in SOD1^G93A^ mouse, a widely used mouse model of ALS (Buck et al., 2017). In contrast, the mitochondrial isoform of SIRT3 is increased in the muscle and spinal cords of SOD1^G93A^ mice (Salvatori et al., 2017). SIRT3 expression attenuates mitochondrial fragmentation and cell death in neurons from SOD1^G93A^ mice (Song et al., 2013). Although inhibition of SIRT2 is thought to have beneficial effects on neurodegenerative diseases such as PD, the deletion of SIRT2 did not appear to effect the disease course of SOD1^G93A^ mice (Taes et al., 2013). Additional studies will be invaluable to unravel the role of other sirtuins in ALS.

### SIRT1 Activator and Clinical Trials

Since accumulating studies have demonstrated the protective effects of SIRT1 against neurodegenerative diseases, potent SIRT1 activators are currently in clinical trials^1^. Clinical trials that are both underway and have been completed have used various proprietary formulations of resveratrol (i.e., SRT501) to treat AD. In one study, treating AD subjects with resveratrol for 52 weeks stabilized the progressive decline in CSF Aβ40 and plasma Aβ40 compared to placebo (Turner et al., 2015). Resveratrol decreased CSF MMP9 and induced adaptive immunity (Moussa et al., 2017), suggesting that resveratrol is beneficial for AD subjects. In addition, there is currently a clinical trial testing resveratrol in HD subjects^2^. However, since SIRT1 is expressed in various tissues, the risk of adverse effects could be considered. To ensure its safety, additional resveratrol and other SIRT1 activator clinical studies are warranted.

## Conclusion

Sirtuins mediate diverse functions in the CNS. The evidence of the beneficial effects of SIRT1 obtained from animal models and human studies imply that SIRT1 activation can be a potential therapeutic treatment for neurodegenerative diseases. Neuronal degeneration in various traumatic injury and neurological disorders, such as SCI, stroke, and AD, is often accompanied by inflammation. It has been reported that activation of SIRT1 also contributes to the suppression of inflammatory responses. Therefore, SIRT1 activation would be a unique strategy in that it is able to control both neurons and inflammatory cells. However, there have also been reports in several animal models of neurodegenerative diseases that SIRT1 activation does not have neuroprotective effects. These conflicting results may be due to a number of factors, including the cell-type-specific alteration of SIRT1 expression following injury.

Understanding the more detailed molecular mechanisms of sirtuins in regulating neuroprotection and degeneration as well as the precise expression patterns of sirtuins following neuronal pathology will contribute to the development of novel anti-neurodegenerative therapeutics. For example, combination treatment with SIRT1 activators and NAD may provide a synergetic strategy that contributes to neuroprotection.

The observation that SIRT2 activity promotes neurodegeneration in a PD model suggests that sirtuins have family-dependent functions. Further studies linking neurodegenerative diseases and sirtuin members, especially SIRT4, 5, and 7, will be helpful to reveal the tissue or disease-specificity of the role of sirtuins. Highly selective compounds targeting specific sirtuins may therefore serve as attractive candidates for a variety of neurological conditions.

## Author Contributions

All authors listed have made a substantial, direct and intellectual contribution to the work, and approved it for publication.

## Conflict of Interest Statement

The authors declare that the research was conducted in the absence of any commercial or financial relationships that could be construed as a potential conflict of interest.

## Footnotes

1 https://clinicaltrials.gov/ct2/results?cond=&term=resveratrol&cntry=&state=&city=&dist=

1 https://clinicaltrials.gov/ct2/results?cond=Huntington+Disease&term=resveratrol&cntry=&state=&city=&dist=
